# A revolving algae biofilm based photosynthetic microbial fuel cell for simultaneous energy recovery, pollutants removal, and algae production

**DOI:** 10.3389/fmicb.2022.990807

**Published:** 2022-10-10

**Authors:** Huichao Zhang, Qian Yan, Zhongyi An, Zhiyou Wen

**Affiliations:** ^1^School of Civil Engineering, Yantai University, Yantai, China; ^2^Department of Food Science and Human Nutrition, Iowa State University, Ames, IA, United States

**Keywords:** microbial fuel cell, algae biofilm, biomass production, pollutant removal, photosynthetic biocathode

## Abstract

Photosynthetic microbial fuel cell (PMFC) based on algal cathode can integrate of wastewater treatment with microalgal biomass production. However, both the traditional suspended algae and the immobilized algae cathode systems have the problems of high cost caused by Pt catalyst and ion-exchange membrane. In this work, a new equipment for membrane-free PMFC is reported based on the optimization of the most expensive MFC components: the separator and the cathode. Using a revolving algae-bacteria biofilm cathode in a photosynthetic membrane-free microbial fuel cell (RAB-MFC) can obtain pollutants removal and algal biomass production as well as electrons generation. The highest chemical oxygen demand (COD) removal rates of the anode and cathode chambers reached 93.5 ± 2.6% and 95.8% ± 0.8%, respectively. The ammonia removal efficiency in anode and cathode chambers was 91.1 ± 1.3% and 98.0 ± 0.6%, respectively, corresponding to an ammonia removal rate of 0.92 ± 0.02 mg/L/h. The maximum current density and power density were 136.1 mA/m^2^ and 33.1 mW/m^2^. The average biomass production of algae biofilm was higher than 30 g/m^2^. The 18S rDNA sequencing analysis the eukaryotic community and revealed high operational taxonomic units (OTUs) of Chlorophyta (44.43%) was dominant phyla with low COD level, while Ciliophora (54.36%) replaced Chlorophyta as the dominant phyla when COD increased. 16S rDNA high-throughput sequencing revealed that biofilms on the cathode contained a variety of prokaryote taxa, including Proteobacteria, Bacteroidota, Firmicutes, while there was only 0.23–0.26% photosynthesizing prokaryote found in the cathode biofilm. Collectively, this work demonstrated that RAB can be used as a bio-cathode in PMFC for pollutants removal from wastewater as well as electricity generation.

## Introduction

Microbial fuel cells (MFCs) are bioelectrochemical reaction devices that can harvest the electric energy generated by special microorganisms in anaerobic respiration process (Feng et al., 2008). A typical MFC contains two chambers, an anaerobic anode chamber and an aerobic cathode chamber separated by a proton exchange membrane (PEM) (Zeng et al., 2010; Deka et al., 2022). Microbial respiration takes place in the anode chamber, and electrons are generated and transmitted to the cathode chamber through an external circuit. The electrons reached to the cathode chamber through the external circuit where it reduces the electron acceptor present while protons produced at the anode are exchanged to the cathode through a membrane separator (Chang et al., 2004; Zhao et al., 2006; Wei et al., 2012; Mathuriya and Yakhmi, 2014) or through the electrolyte in a membraneless cell (Taşkan et al., 2021). Oxygen is an economical and environmentally friendly electron acceptor noble metal platinum (Pt) is commonly used as a catalyst in cathode chamber for oxygen-reduction reaction (Jiang et al., 2019). The utilization of Pt-based catalysts on cathode surface is not only costly, but also easily contaminated by the organic and inorganic fouling (Kolajo et al., 2022). The contamination of Pt catalyst is the main reason that leading to system failure after long-time operation (Liew et al., 2014). Furthermore, the membrane settled between the anode and cathode are estimated to account to 47% cost in lab-scale MFCs and over 60% of the material cost in large-scale application (Rodríguez et al., 2021).

As an alternative for the Pt-based catalyst, microalgae have been explored as a biocatalyst in the cathode chamber of MFC (Reddy et al., 2019). When algal culture is integrated with MFC system, algal cells produce oxygen as electron acceptors for the redox reaction (Mohamed et al., 2020). In addition, algae-based MFCs can use wastewater for algal growth (Wang et al., 2010; Wu et al., 2014) and provide the benefit of removing nutrients from wastewater. The algal biomass produced in MFCs can also be developed into value-added products (Arora et al., 2021).

In algae-based MFC systems, algal cells in the cathode chamber are commonly grown in suspension (Powell et al., 2009; Colombo et al., 2017; Nguyen et al., 2017). Due to the inherent limitations of suspended algal cultivation system such as light limitation and low cell density, the cathode electron reduction rate of algae-based MFCs is very low, which limits the efficiency of the whole MFC system. For example, Powell et al. (2009) investigated the use of *Chlorella vulgaris* as a biocathode in a MFC, the ultimate voltage was only 70 mV, corresponding to a power density of 2.7 mW/m^2^.

Compared to suspended algal cultures, algal biofilm systems have emerged as an innovative technology in MFCs (Zhou et al., 2012; Jadhav et al., 2017). The algal biofilm based MFCs has been reported to increase dissolved oxygen and decrease cathodic charge transfer resistance (Mohan et al., 2014; Jadhav et al., 2017). For example, an immobilized *Chlorella vulgaris* MFC produced an 88% higher power density than the system with the suspended culture of the same strain (Zhou et al., 2012).

Among various algal biofilm cultures, a revolving algae biofilm (RAB) system has been developed and proven as an effective system for wastewater treatment (Gross et al., 2013, 2015; Zhao et al., 2018; Zhou et al., 2018). In the RAB system, algae and bacteria stick to a vertically oriented belt and forming a biofilm which rotates between the liquid reservoir and the gas phase. The algal cells absorb nutrients from the liquid phase while receiving sunlight and carbon dioxide from the gas phase. The RAB reactor occupies a much smaller footprint but generates 5–10 times more biomass than the suspended cultivation systems (Zhao et al., 2018). Furthermore, algal cells in the RAB can be harvested through scrapping the biofilm, therefore, the cell harvesting costs can be significantly reduced (Gross et al., 2013, 2015). In this context, recycled algal biomass can be used to produce high value-added products at a lower cost.

When RAB system is integrated with MFC, it is hypothesized that the attachment of algal cells on the electrode surface affects oxygen diffusion, MFC performance and corrosion. The RAB reactor produces oxygen at a much faster rate than the suspended system, which is highly desirable as production rate of the electron reduction receptors can greatly improve the performance of algae-MFC system (Saba et al., 2017). The aim of this study is to explore the feasibility of using RAB system as the bio-cathode and evaluate the enhancement of electricity generation performance in MFC. Meanwhile, the nutrient removal performance by the revolving algae-bacteria biofilm cathode in a photosynthetic membrane-free microbial fuel cell (RAB-MFC) system was studied to evaluate the feasibility of using this system as an effective wastewater treatment technology.

## Materials and methods

### Design and construction of the revolving algae-bacteria biofilm cathode in a photosynthetic membrane-free microbial fuel cell system

**FIGURE 1:**
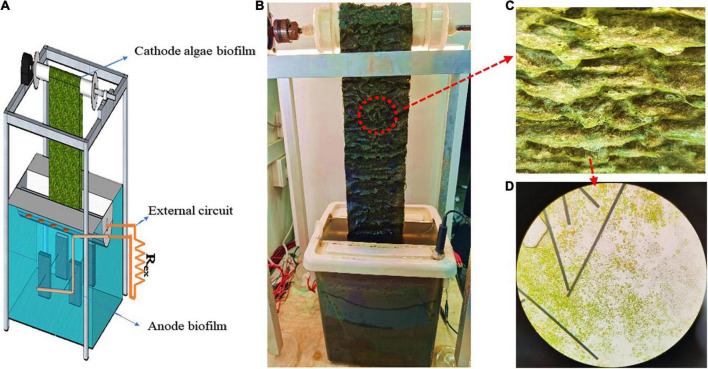
Schematics of the RAB-MFC system **(A)**; the photo of RAB-MFC system **(B)**; algae biofilm on the carbon cloth **(C)**; and the micrograph of algae biofilm (10 × 40) **(D)**.

An integrated RAB-MFC system was designed and constructed. As shown in Figures 1A,B, the system consists of three sections, an anode section, a cathode section and an external circuit. The rectangular anode chamber (20 cm × 26 cm × 22 cm, 12-L working volume) was located at the bottom of the system. An anode electrode was made of a carbon felt (9 cm × 5 cm × 1 cm) which was immersed into the chamber; titanium wires (1 mm in diameter) were embedded into the felt leading the electrons to the external circuit. During the operation, the anode chamber was covered to maintain dark and prevent algal growth.

The cathode section was a modified RAB system. The revolving belt was made of carbon cloth (150 mm wide × 600 mm length) sandwiched with polyester web sheets; the carbon cloth accepted electrons from the external circuit while the polyester web provided mechanical strength. The belt was vertically oriented and revolving between the top actuated driveshaft and the bottom passive shaft. Approximately 10% of the belt surface area (together with the passive shaft) was submerged into a liquid reservoir while the remaining belt was exposed to the air. In the air phase, the algal biofilm was continuously illuminated under artificial irradiation at 130 μmol/m^2^/s. The liquid reservoir was made from a half-cylindrical pipe (100 mm diameter and 260 mm length) with a working volume of 1.5 L.

To integrate the anode and cathode chambers, wires and resistances were used to connect the anode carbon felt and the cathode belt to create an external circuit (Figure 1A), a slot opening was cut at the top of the anode chamber and the bottom of the cathode liquid reservoir was sitting onto this slot. To enable of the exchange of liquids and ions between the anode and cathode chambers, the bottom of the cathode liquid reservoir was cut with two rows of holes (5 holes per row and 5 mm diameter each hole) which were evenly distributed (every 3 cm). Prior to use, the carbon felt in the anode chamber and the sandwiched belt in the cathode reservoir were washed with 1 M HCl for 48 h and rinsed with deionized water to remove trace metals.

### Microorganisms

Anaerobic sludge collected from an anaerobic digester at Water Pollution Control Plant in Ames, Iowa, USA, was used as the start culture for the anode chamber. The synthesis wastewater for the RAB-MFC system was prepared by dissolving sodium acetate (1.64 g/L or 3.28 g/L COD equivalent) in a nutrient solution containing (per liter) 4.4 g KH_2_PO_4_, 3.4 g K_2_HPO_4_⋅3H_2_O, 0.5 g NH_4_Cl, 0.1 g MgCl_2_⋅6H_2_O, 0.1 g CaCl_2_⋅2H_2_O, 0.1 g yeast extract, and 10 mL trace mineral metals solution (Wen et al., 2012).

### Operation of the revolving algae-bacteria biofilm cathode in a photosynthetic membrane-free microbial fuel cell system

The operation of the RAB-MFC system was initiated with establishing the biofilm in both the anode electrode (carbon felt) and the cathode electrode (the sandwiched belt). During this stage, the connecting holes between the anode chamber and the cathode reservoir were blocked and the external circuit was disconnected. The anaerobic activated sludge was first acclimated in dark for 7 days by feeding anolyte. The acclimated liquid was then mixed with fresh anode solution at 1:9 ratio (v/v) and loaded into in the anode chamber. Bacteria biofilm was gradually formed on the surface of the submerged felt in the chamber by observing the carbon mat with the naked eye and the microscope.

To establish algal biofilm on the surface of the sandwiched belt in the cathode section, microalgal seed culture prepared from a raceway pond (1,000-L working volume) at the Algal Production Facility at Iowa State University Research Farm (Boone, IA, USA) was mixed with Bold’s Basal Medium (BBM) (1:2 ratio, v/v) and loaded into the cathode reservoir. The belt was started revolving at 10 revolutions per minute for 7 days. During this period, suspended algae gradually attached on the belt surface. To facilitate the attachment, 0.5 L liquid from the reservoir was replaced with fresh seed/BBM mixture every two days to supplement nutrient for algae.

After the biofilms were established in both the anode chamber and cathode belt. The holes connecting the anode chamber and cathode reservoir were unblocked. The external circuit was also connected with external resistance 1,000 Ω. The system was operated with semi-continuous mode. The anolyte and catholyte were discharged and fresh solution were added into the chambers every 6–7 days. Meanwhile, every 48 h, 0.5 L water was added into an anode chamber which overflew to the cathode chamber to compensate water evaporative loss. The algal cells on the cathode belt was harvested by scraping the biofilm every two days with area 4 cm^2^ and cleaned it up every two liquid exchange cycles. The weight of biomass was measured after drying for 2 h at 105°C. The operation of the RAB-MFC system was considered successfully established when the voltage of the external resistance (1,000 Ω) reached 250 mV with a repeatable highest value after two liquid discharge cycles. During the operation, two COD concentrations (1,200 and 2,400 mg/L) of anolyte was studied. The hydraulic retention time (HRT) was set as 6- and 7-day for the 1,200 and 2,400 mg/L anolyte, respectively. Two identical RAB-MFC systems were operated at ambient temperature (25 ± 2°C) at each condition. A series of external resistances with different values (ranging from 10 Ω to 10 kΩ) were used to determine the power density curve and polarization curve (Wen et al., 2012).

### Determination of electrical properties of the revolving algae-bacteria biofilm cathode in a photosynthetic membrane-free microbial fuel cell system

The voltage (*E*) across the circuit resistance (*Re*) of the RAB-MFC system was monitored with a voltmeter (TRMS 6000, AstroAl, USA). The current (*I*) through the electrical circuit was determined as


(1) $$I=\frac{E}{R_{e}}$$

The current density (*I*_*den*_) and power density (*W*_*den*_) were determined as


(2) $$I_{d\,e\,n}=\frac{I}{S}$$

(3) $$W_{d\,e\,n}=I\times \frac{E}{V_{a\,n}}$$

where *S* is projection area of the anode electrode, *V*_*an*_ is the active volume of anode chamber.

### Analyses

Chemical oxygen demand (COD) was determined using Hach kits (TNT 822). Total nitrogen (TN) was analyzed using Hach kits (TNT880). Ammonium concentration was analyzed based on the salicylate method (Hach Method 10023). Nitrate concentration was measured based on APHA method 4500 (Arnold and Lenore, 1992). pH of samples was measured using a pH meter (HANNA, HI8424, Italy). The conductivity of the anolyte and catholyte were determined with a hand-held conductivity meter (Qiwei, DDB-12H, China). A light microscopy (Jiangnan, J-112, China) was used to observe the composition of the cathode biofilm. The anaerobic and aerobic effluent was taken from anodic chamber and cathodic chamber, respectively.

The nutrients (ammonia, COD, TN) removal efficiencies (*E*) were determined as


(4) $$E=\left(\frac{C_{i\,n}-C_{e\,f\,f}}{C_{i\,n}}\right)\times 100\%$$

where *C*_*in*_ and *C*_*eff*_ are the concentrations of the nutrients in the influent and effluent, respectively.

The nutrient removal rate (*R*, mg/L/h) of each nutrient were determined as


(5) $$R=\frac{\left(C_{i\,n}-C_{e\,f\,f-a\,n}\right)\times V_{a\,n}+\left(C_{i\,n}-C_{e\,f\,f-c\,a\,t}\right)\times V_{c\,a\,t}}{t}$$

where *C*_*eff–an*_ and *C*_*eff–cat*_ are the concentrations of the nutrients in anode and cathode effluent, respectively. *V*_*an*_ and *V*_*cat*_ are volume of anode and cathode chamber, respectively.

### Microbial diversity measurement

#### DNA extraction, PCR, and high-throughput sequencing

Microbial communities of biofilms in the RAB reactors were characterized by Illumina high-throughput sequencing. Genomic DNA was extracted and then analyzed by 1% agarose gel electrophoresis. 18S rRNA gene sequencing of V4 region for eukaryota was amplified with the primers 528F (5′-GCGGTAATTCCAGCTCCAA-3′) and 706R (5′-AATCCRAGAATTTCACCTCT-3′) (Zhou et al., 2018). For 16S rRNA sequencing of the V4 and V5 regions for bacteria, 515F (5′-GTGCCAGCMGCCGCGGTAA-3′) and 907R (5′-CCGTCAATTCCTTTG AGTTT-3′) were used as primers in amplifying.

#### Analysis of microbial community diversity and richness

Operational taxonomic units (OTUs) classify closely related individuals (e.g., strains, genera, or species) based on the phylogenetic or population genetics. Clustering was necessary to obtain the number of bacteria and genera present in the sample based on the sequencing results. All sequences were divided into OTUs according to their degree of similarity, and a threshold similarity of 97% is typically used in the biological analyses. The community composition of each sample was determined at the kingdom, phylum, class, order, family, genus, and species levels. The Silva, Ribosomal Database Project (RDP), and Greengene databases were used for the comparison. Functional gene repository (FGR) and RDP were used for the functional gene analysis, based on the GenBank functional gene database. The Qiime platform was used with a confidence threshold of 0.7 for the RDP classifier.

## Results and discussion

### Electricity generation of the revolving algae-bacteria biofilm cathode in a photosynthetic membrane-free microbial fuel cell system

#### Voltage change

**FIGURE 2:**
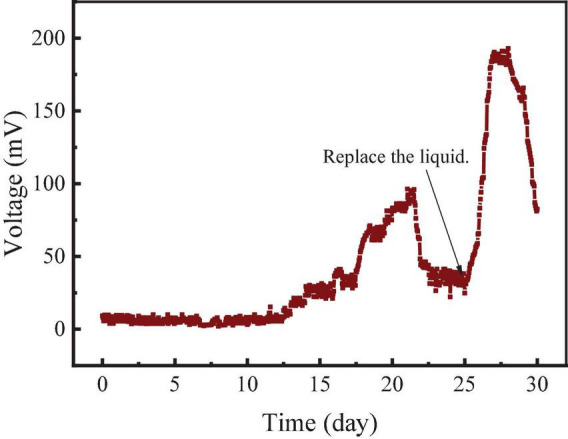
Voltage of the revolving algae-bacteria biofilm cathode in a photosynthetic membrane-free microbial fuel cell (RAB-MFC) system during the initial biofilm establishment stage.

The startup stage of the RAB-MFC system consisted of two processes, the establishment of the anodic electrochemical biofilm and cathodic algae-biofilm. As shown in Figure 1, a layer of green algae biofilm was observed on the surface of the cathode electrode (belt) in about 15 days. The algal biofilm mainly composed of *Chlorella* cells (Figures 1C,D). The voltage of the RAB-MFC system during the startup stage was shown in Figure 2. The system started to show electrochemical activity after 12 days operation, voltage increased from zero to 96 mV at day 21, and then decreased due to the exhaustion of the organic carbon (acetate) in the anode chamber. At day 25, the anode and cathode chambers were replaced with fresh anode solution and BBW, respectively, resulting in a rapid voltage recovery. The voltage reached to the peak value of 192.6 mV at day 27, corresponding a current density 42.8 mA/m^2^ power density of 8.24 mW/m^2^. The voltage dropped again due to the consumption of organic matter in the anolyte.

**FIGURE 3:**
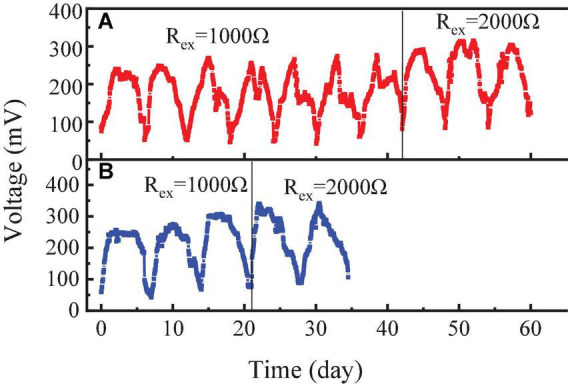
Voltages of the revolving algae-bacteria biofilm cathode in a photosynthetic membrane-free microbial fuel cell (RAB-MFC) system during the semi-continuous operation with the influent organic strength at chemical oxygen demand (COD) 1,200 mg/L **(A)** and COD 2,400 mg/L **(B)**, respectively.

After the initial electrochemical activity was established, the RAB-MFC system was switched to a semi-continuous operation by feeding the anode chamber with influent. The voltage output are shown in Figure 3. The highest voltage reached to 315 mV with an external resistance of 1,000 Ω, equivalent to current density of 59.8 mA/m^2^ and power density of 16.1 mW/m^2^. In a previous study, the *Cladophora* sp. was used to provide oxygen to the cathode of the photosynthetic biocathode membrane-less microbial fuel cell, which produced a voltage of 203.61 mV with 1,000 Ω external resistance (Taşkan and Taşkan, 2022). When the external resistance increased from 1,000 to 2,000 Ω, the external resistance voltage increased from 315 to 350 mV.

Figure 3 also shows that at an external resistance of 1,000 Ω, increasing COD from 1,200 to 2,400 mg/L did not cause significant voltage change. However, with external resistance 2,000 Ω, the voltage of the system significantly (*P* < 0.05) increased with enhancing the COD level of the feed from 1,200 to 2,400 mg/L. Higher influent COD concentration also affected the algal growth. After four consecutive feeding of influent with 2,400 mg/L COD, the color of the biofilm on the cathode surface changed from green to brown, indicating the taking over of bacteria in the microbial consortium in the biofilm. Nguyen et al. (2017) also reported that lower COD concentration was more conducive to the release of oxygen by algae with a maximum MFC voltage of 300 ± 11 mV being obtained with 5% leachate. In another study, increasing influent COD concentration from 2,500 to 5,000 mg/L led to a significant voltage decline in MFC (Mohan et al., 2014).

A variety of algae based MFC systems have been developed with the ion exchange membranes being dominantly used to connect anode and cathode chamber (Arun et al., 2020). In this work, the anode and cathode chambers was connected with the connection holes, and thus, cost of using expensive membrane was avoided. On the other hand, the connection holes may cause the oxygen diffusion from cathode solution into the anode chamber. As a result, part of the electrons in anode chamber can be consumed by oxygen diffused from the cathode chamber. Therefore, optimizing the configuration of the connecting holes (such as diameter, numbers, and the layout) between anode and cathode is needed in the future work so that the diffusion of oxygen can be controlled and the output voltage can be improved.

#### Power density

**FIGURE 4:**
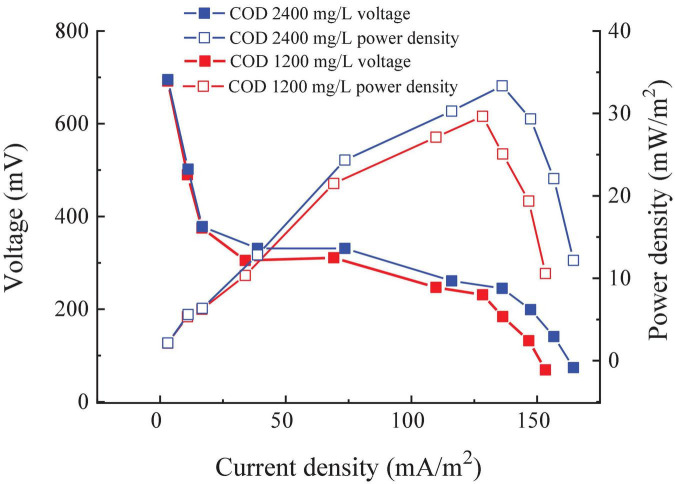
Power density and polarization curve of the revolving algae-bacteria biofilm cathode in a photosynthetic membrane-free microbial fuel cell (RAB-MFC) system.

The external resistance of the RAB-MFC system was modified to obtain the power density of the system. As shown in Figure 4, the open-circuit voltage (OCV) of the RAB-MFC system was recorded as 695 and 700 mV at the COD of 1,200 and 2,400 mg/L, respectively, a level higher than the OCV of H-type MFC integrated with suspended algae culture as the catholyte (570 mV) (Mohamed et al., 2020). The anode potential of different types MFCs is basically similar (Logan et al., 2006). The OCV difference between H-type MFC and RAB-MFC system as considered due to the higher elevated potential of algal-biofilm cathode in the RAB-MFC system. Higher cathodic potentials in RAB-MFC revealed that electrons transferred from external circuits rapidly bound to oxygen produced by algae biofilm. Figure 4 also shows that the maximum power density reached 33.07 mW/m^2^ with influent COD of 2,400 mg/L, at this time the external resistance is 400 Ω. Based on the law of voltammetry, the highest power density is obtained when the external resistance equals to the internal resistance (Liu et al., 2004, 2005; Logan et al., 2006; Logan, 2009), therefore, the result in Figure 4 indicates that the internal resistance of the RAB-MFC system was 400 Ω.

By comparing the OCV with connect circuit with external 2,000 Ω, we can see that voltage dropped by nearly half. This was mainly due to the high internal resistance in the RAB-MFC system, which consumed part of the voltage. Mohamed et al. (2020) reported that the internal resistances of algae MFC cathode modified with *Synechococcus* sp. or *Chlorococcum* sp. were 448 and 512 Ω, respectively, which was comparable to the internal resistance of the RAB-MFC system obtained at this work. However, the internal resistance of the RAB-MFC system was higher than most MFCs with potassium ferricyanide as the chemical or oxygen cathode and Pt as a catalyst, ranging from 20 to 100 Ω (Fan et al., 2008). It has been reported that increasing the distance between the anode and cathode resulted in an increased internal resistance (Manohar and Mansfeld, 2009), which can be the reason for higher resistance of the RAB-MFC reported here. From a sustainability perspective, however, the RAB-MFC system is advantageous as chemical catholyte cannot last for long periods and the cathode electrolyte requires regular replacement (Zhang et al., 2016).

#### Comparison of the revolving algae-bacteria biofilm cathode in a photosynthetic membrane-free microbial fuel cell system with other algae-based microbial fuel cell systems

A comparison of the performances of different microalgae-based MFC systems is presented in Table 1. Among various systems, the RAB-MFC showed a medium power generation capacity. Nevertheless, the COD removal efficiency and scale of the RAB-MFC system were higher than other systems. Additionally, the RAB-MFC system can be easily scaled up for its cathode area by increasing the length of belt, which is highly desirable for practical application for wastewater treatment (Zhao et al., 2018).

**TABLE 1:** Comparison of algae-based cathode MFC system performance.

Types of MFC	Separator	Function algae species	Maximum power density (mW/m^2^)	Effective anode volume (L)	COD removal efficiency	Ref.
H-type Two chambers	Cation exchange membrane	*Chlorella vulgaris*	8.79	0.4	84.8%	Zhou et al., 2012
Two chambers sediment MFC	Multi-walled carbon nanotubes	*Chlorella vulgaris*	38	0.65	NG	Wang et al., 2014
Sediment microbial carbon-capture cell		*Chlorella vulgaris*	22.19	2.54	77.6 ± 2.1%	Neethu and Ghangrekar, 2017
H-type Two chambers	Cation exchange membrane	*Chlorella sp. QB-102*	36.4*	0.25	NG	Zhang et al., 2018
H-type Two chambers	Proton exchange membrane	*Chlorococcum* sp. *Synechococcus* sp.	30.2 41.5	0.25 0.25	69.4% 73.5%	Mohamed et al., 2020
RAB-MFC	Membrane-free (connection holes)	Algae biofilm	33.1	12.5	93.5 ± 2.6%	This study

*Modificatory Ni-rGO cathode was used in this study.

Photosynthetic algae microbial fuel cells are currently used for generation of the electrical power, fixation of carbon dioxide, and removal of organic or nutrient matter (Baicha et al., 2016; Nguyen et al., 2017). Velasquez-Orta et al. (2009) used microalgae and macrophyte in the anode of signal MFC chamber for energy recovery, COD degradation, and power generation. Their results revealed the usefulness of algae as a renewable source of electrical power production in MFCs. Ndayisenga et al. (2018) examined the role of microalgae *Chlorella* biomass as the sole electron donor in double-chamber H-type MFC anodes. Compared to acetate as anolyte, the power generation and COD removal capability of algal-fed MFC were similar. The maximum power generation, columbic efficiency and COD removal rate reached 1.07 W/m^2^, 61.5 and 65.2%, respectively. As a result, future biomass from RAB-MFC algal biofilms might also be utilized in anodes as fuel to yield self-sufficient systems.

Compared with traditional H-type MFC, the RAB-MFC system used algal biofilm as the cathode, and oxygen produced by microalgae can improve the electron transfer efficiency. In turn, electrons transferred from the anode can promote the growth of biomass of the cathode algae. The whole system also showed high pollutant removal capability.

### Pollutants removal performance

#### Chemical oxygen demand removal of the revolving algae-bacteria biofilm cathode in a photosynthetic membrane-free microbial fuel cell system

**FIGURE 5:**
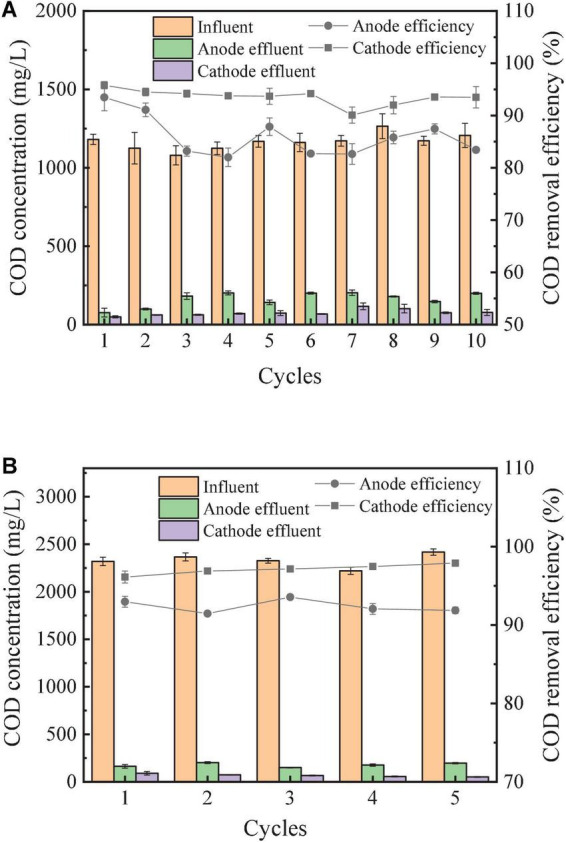
COD removal efficiency in semi-continuous operation of the RAB-MFC system. Chemical oxygen demand (COD) of feed was 1,200 mg/L **(A)**, and 2,400 mg/L **(B)**.

The COD removal by the both the anode and cathode chambers of the RAB-MFC system were presented in Figure 5. The highest COD removal rates of the anode and cathode chambers with the influent COD of 1,200 mg/L reached 93.5 ± 2.6% and 95.8% ± 0.8%, respectively (Figure 5A). In the cathode chamber, COD can be further removed by the microbial consortium deposit on the cathode electrode surface. The presence of bacteria, cyanobacteria, and eukaryotic microorganisms has been reported in the RAB system (Zhou et al., 2018). Most of these microorganisms such as Acidobacteria (*Terriglobus*), algae (*Eustigmatophyceae*, *Nannochloropsis*), and Chlorophyta (*Acutodesmus*) can consume organic carbon from the catholyte (Zhou et al., 2018).

As influent COD increased to 2,400 mg/L, the COD removal rate reached 95% in the cathode chamber (Figure 5B). The continuous operation of the RAB-MFC system at this influent COD level was stopped when the color of the biofilm on the cathode surface changed to light brown, indicating the takeover of bacteria in the microbial consortium.

#### Nitrogen removal of the revolving algae-bacteria biofilm cathode in a photosynthetic membrane-free microbial fuel cell system

**FIGURE 6:**
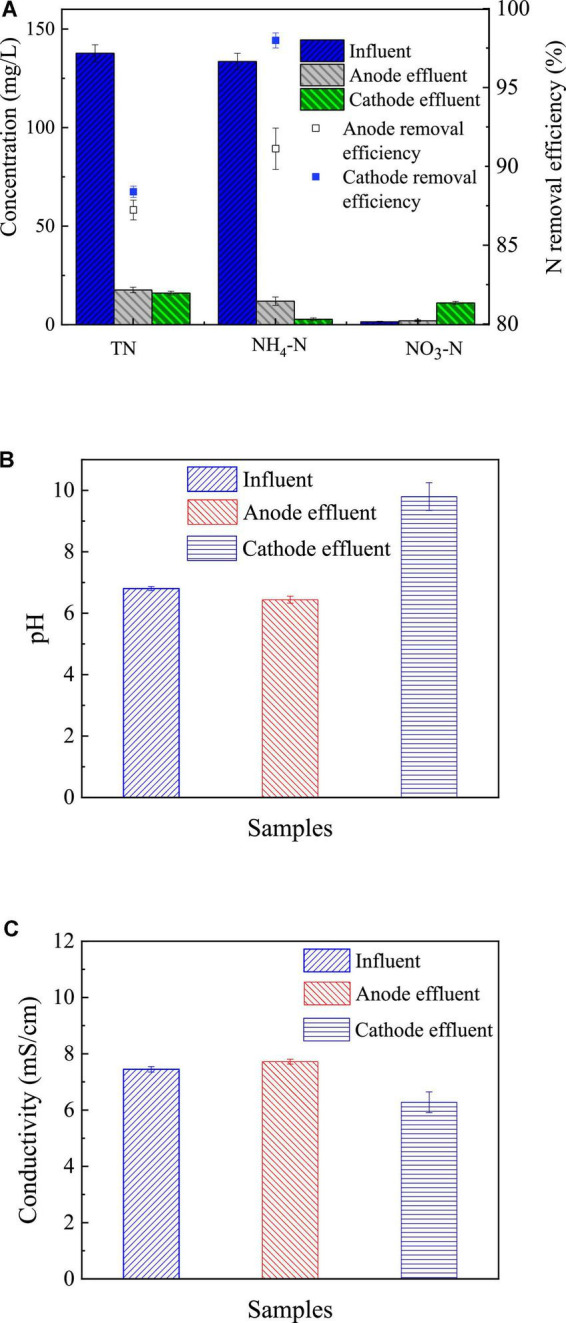
Nitrogen removal in the revolving algae-bacteria biofilm cathode in a photosynthetic membrane-free microbial fuel cell (RAB-MFC) system **(A)**; pH **(B)**; and conductivity **(C)** of influent and anode and cathode effluent of the RAB-MFC. Influent chemical oxygen demand (COD) was 1,200 mg/L.

Nitrogen removal by the RAB-MFC system was also evaluated. As shown in Figure 6A, total nitrogen (TN) concentration decreased from 137.65 ± 4.31 mg/L in the fluent to 17.6 ± 1.41 mg/L in anode effluent and 16 ± 1.00 mg/L in cathode effluent, respectively. While NH_4_-N dropped from 133.5 ± 4.1 mg/L in influent to 11.9 ± 2.1 mg/L and 2.71 ± 0.72 mg/L in the anode and cathode effluents, respectively. Meantime, the concentration of NO_3_-N increased significantly in cathode chamber, from 1.46 mg/L to 11.0 ± 0.85 mg/L, indicating the conversion of ammonium to nitrate by the RAB-MFC system. The removal efficiency of ammonia in the anode and cathode chambers were 91.1 ± 1.3% and 98.0 ± 0.6%, respectively.

The RAB-MFC system also demonstrated an ammonia removal rate of 0.92 ± 0.02 mg/L/h, a level higher than that of a single RAB system (0.64 ± 0.12 mg/L/h) (Zhou et al., 2018). Note that microorganisms at the anode promoted the removal of ammonia due to the anabolism, and anammox may occur in the anode chamber (Min et al., 2005; Domenico et al., 2015).

The ammonia removal efficiency of the RAB-MFC system was also higher than that of the conventional MFC systems. Kim et al. (2008) recorded an ammonia removal efficiency of 60% over 5 days in single-chamber MFC, and 69% in two-chamber MFC over 13 days. The RAB-MFC system removed over 90% of ammonia. Some bacteria, such as nitrifying bacteria, do not consume COD but can oxidize ammonia nitrogen to nitrate nitrogen in algal biofilm (Shen et al., 2019).

Ammonia loss may also be resulted from an elevated pH in the cathode chamber (Figure 6B), which was caused by the photosynthesis of algae, i.e.,


(6) $${\text{H}}^{+}+\mathrm{HC}\,{\text{O}}_{3}^{-}↔C\,{\text{O}}_{2}+{\text{H}}_{2}\,\text{O}$$

According to Eq. (6), during the fixation of carbon dioxide in photosynthesis, H^+^ ions are consumed in the cathode chamber (Chi et al., 2011). Meantime, OH^–^ ions was produced in the cathode during the current generation, i.e.,


(7) $${\text{O}}_{2}+4\,{\text{e}}^{-}+4\,{\text{H}}^{+}\to 2\,{\text{H}}_{2}\,\text{O}$$

(8) $${\text{O}}_{2}+4\,{\text{e}}^{-}+2\,{\text{H}}_{2}\,\text{O}\to 4\,{\text{OH}}^{-}$$

or


(9) $${\text{O}}_{2}+2\,{\text{e}}^{-}+2\,{\text{H}}_{2}\,\text{O}\to {\text{HO}}_{2}^{-}+{\mathrm{OH}}^{-}$$

(10) $${\text{HO}}_{2}^{-}+2\,{\text{e}}^{-}+2\,{\text{H}}_{2}\to 4\,O\,{\text{H}}^{-}$$

In a two-chamber MFC system, an essential way for nitrogen loss from the anode chamber is the diffusion of ammonium ions through the membrane between the anode and cathode chambers (Jang et al., 2004; Kim et al., 2008). In RAB-MFC, the material exchange between two chambers was through the connecting holes, ammonia can quickly transfer from the anode to cathode as there is no membrane-resistance. Membrane-free microbial fuel cells also possessed excellent performances toward COD removal and power generation, and can be easily scaled up due to the simple configuration and smooth operation (Zhu et al., 2011).

Figure 6C shows the changes of the conductivity of the anolyte and catholyte after one cycle. Compared to the influent, the conductivity of the cathode effluent was slightly lower, while the conductivity of the anode effluent slightly increased. This indicates that the ionic strength in the anode solution increases while the ionic strength in the cathode solution decreases compared to the influent. In the anode chamber, the bacteria can utilize acetate and produce carbon dioxide, which may increase the conductivity of the anolyte (Logan, 2009). In the cathode chamber, ammonium ions, bicarbonate and carbonate plasma were absorbed and utilized by algal cells, reducing the ionic strength of cathode solution.

### Biomass production performance

**FIGURE 7:**
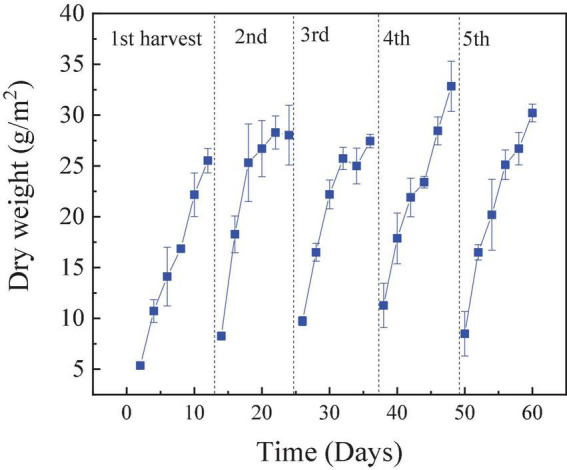
Weight of the dry algae biofilm harvested from the revolving algae-bacteria biofilm cathode in a photosynthetic membrane-free microbial fuel cell (RAB-MFC) cathode reactor. Influent chemical oxygen demand (COD) was 1,200 mg/L.

The RAB-MFC was operated continuously for approximately 6 months, and the biomass curve of the five typical cycles with influent COD 1,200 mg/L is illustrated in Figure 7. As shown in Figure 7, with the addition of a nitrogen source in anolyte, the growth of the algal biofilm in cathodic biofilm was vigorous, and it continued to grow in the later stages of the cycle. The biomass weight of the algae biofilm was greater than 30 g/m^2^. The biomass of the algae biofilm with was higher than that reported in a study using domestic sewage as the cultivation medium (Gross et al., 2013) and another study using polyacrylamide in simulating oil flooding wastewater as N resource (Zhang et al., 2021). The concentration of nitrogen in water directly affects the growth of algal biofilm (Lin et al., 2013; Mathew et al., 2022).

### Bioanalysis

To explore the microorganisms and characteristics of the biofilm community involved in biocathode, high-throughput sequencing was used to analyze prokaryotes and eukaryotes in biofilms from biocathode biofilms with two different initial COD concentrations.

#### Variation in the eukaryota community composition

**FIGURE 8:**
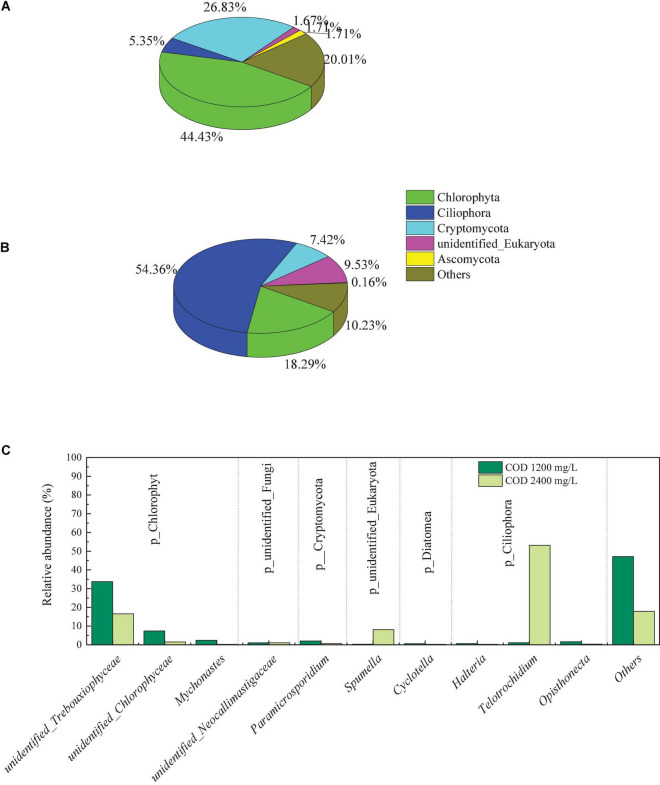
Eukaryotic community abundance at the phylum level in the biofilms on the biocathode of 1,200 mg/L **(A)**, and 2,400 mg/L **(B)** and at the genus level **(C)**.

Changes in the abundance of eukaryote in the biocathode biofilms were detected at the phyla and genus level, as shown in Figure 8. At a COD concentration of 1,200 mg/L, the first three phyla were Chlorophyta, Cryptomycota and Ciliophora, with a relative abundance of 44.43, 26.83, and 5.35%, respectively. However, when COD concentration was increased to 2,400 mg/L, Ciliophora was the dominant phylum, with a relative abundance of 54.36%. The Chlorophyta lost its advantage, the relative abundance of it dropped from 44.43 to 18.29%. The abundance of Cryptomycota showed the same trend, dropped from 26.83 to 7.42%. These results indicated that when the concentration of COD in the solution increased, the proportion of autotrophic eukaryotes in the biofilm decreases, while the proportion of heterotrophic eukaryotes increases. *Chlorella* sp. from Chlorophyta phylum is the potential strain for microalgal-assisted MFCs and Utilization of it in MFCs can efficiently reduce TN, TP, and COD (Sharma et al., 2022).

On the genus level, *unidentified_Trebouxiophyceae* (33.75%) and *Telotrochidium* (53.13%) was the dominant genus in biofilms of COD 1,200 mg/L and 2,400 mg/L, respectively. The relative abundance of *unidentified_Trebouxiophyceae* in cathodic biofilm dropped to 16.53% with COD increasing. *Unidentified_Trebouxiophyceae* are members of the phylum Chlorophyta while *Telotrochidium* are members of the phylum Ciliophora. The presence of green algae in the cathode contributed to the production of oxygen, and the algae can increase the pH of the culture medium (Sharma et al., 2021).

#### Variation in the prokaryotic community

**FIGURE 9:**
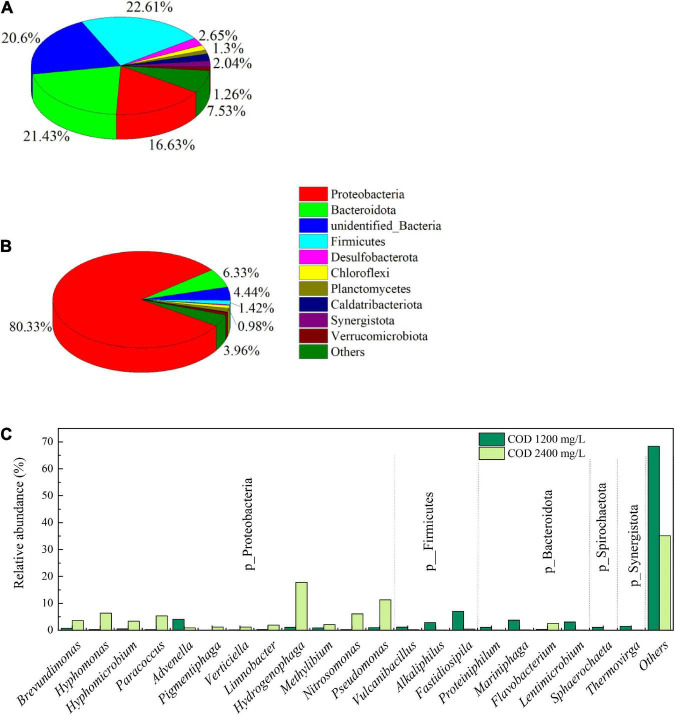
Prokaryotic community abundance at the phylum level in the biofilms on the biocathode of 1,200 mg/L **(A)**, and 2,400 mg/L **(B)** and at the genus level **(C)**.

Figure 9 summarizes the relative abundant of procaryotic organism at their phyla and genus level. On the phyla level, the community in biofilm samples with COD 1,200 mg/L was more diverse than that of COD 2,400 mg/L. There were three phyla with high abundance in the sample of biofilm with COD 1,200 mg/L, Proteobacteria, Bacteroidota and Firmicutes, ranged between 16.33 and 22.2%. When COD increased to 2,400 mg/L, the relative abundance of Proteobacteria occupied an absolute dominant position, with a relative abundance of 80.33%. The relative abundance of Bacteroidota and Firmicutes were only 6.33 and 1.42% with higher COD concentration. The bacteria containing chlorophyll and capable of photosynthesis, such as Cyanobacteria, were only 0.23 and 0.26% in cathode biofilm samples with COD 1,200 and 2,400 mg/L, respectively. This suggests that the microorganisms capable of producing oxygen at the cathode biofilm are mainly eukaryotic algae.

The most abundant genera in the biocathode biofilm of COD 1,200 mg/L were *Fastidiosipila* (7.06%), followed by *Advenella* (4.12%), *Mariniphaga* (3.74%), *Lentimicrobium* (3.06%), and *Alkaliphilus* (2.89%). In a couple of previous reports, *Fastidiosipila* genus is involved in the metabolism of ammonia and organic matter, activated carbon and biochar could improve the relative abundance of *Fastidiosipila* (Li et al., 2022; Wang et al., 2022). The growth of *Fastidiosipila* in cathode biofilm may be due to the active carbon fiber as the substrate of cathode. When COD was increased, the community structure of biocathode biofilm changed greatly, and the previously dominant bacterial genera were replaced by new dominant bacterial genera. The *Hydrogenophaga* was dominant genus with a relative abundance 17.94%, followed by *Pseudomonas* (11.28%), *Hyphomonas* 6.34%), *Nitrosomonas* (6.06%), and *Paracoccus* (5.31%). *Hydrogenophaga* is able to degrade alginate as a sole carbon source under aerobic conditions (Yamaguchi et al., 2019). The genus of *Nitrosomonas* can convert ammonium to nitrite, which will help to remove ammonia from the cathode solution (Park et al., 2022).

## Conclusion

This work demonstrates that RAB system can be successfully used in a membraneless photosynthetic microbial fuel cell to supply sufficient oxygen for electrons reduction. The RAB-MFC system showed a superior performance of pollutants removal, electrons also be recovery in this process. The inner resistance of the RAB-MFC system was about 400 Ω while a maximum power density of 33.07 mW/m^2^. The COD and ammonia removal efficiency in RAB-MFC system was 95.8% ± 0.8% and 98.0 ± 0.6% with initial COD concentration 1,200 mg/L, respectively. The 18S rDNA and 16S rDNA sequencing analysis the eukaryotic and prokaryote community, respect and revealed high OTUs of Chlorophyta (44.43%) was dominant phyla with low COD level, while Ciliophora (54.36%) replaced Chlorophyta as the dominant phyla when COD increased. 16S rDNA high-throughput sequencing revealed that biofilms on the cathode contained a variety of prokaryote taxa, including Proteobacteria, Bacteroidota, Firmicutes, while there was only 0.23–0.26% photosynthesizing prokaryote found in the cathode biofilm.

The membrane-free RAB-MFC system enhanced substance exchange between anode chamber and cathode chamber and can be practically scaled up.

## Data availability statement

The raw data supporting the conclusions of this article will be made available by the authors, without undue reservation.

## Author contributions

HZ: conceptualization and writing—original draft preparation. QY: writing—reviewing and editing. ZA: funding acquisition. ZW: supervision and project administration. All authors contributed to the article and approved the submitted version.
